# Selective Estrogen Receptor Modulators: A Potential Option For Non-Binary Gender-Affirming Hormonal Care?

**DOI:** 10.3389/fendo.2021.701364

**Published:** 2021-06-18

**Authors:** Jane Y. Xu, Michele A. O’Connell, Lauren Notini, Ada S. Cheung, Sav Zwickl, Ken C. Pang

**Affiliations:** ^1^ Geisel School of Medicine, Dartmouth College, Hanover, NH, United States; ^2^ Clinical Sciences and Genetics Themes, Murdoch Children’s Research Institute, Parkville, VIC, Australia; ^3^ Department of Adolescent Medicine, Royal Children’s Hospital, Melbourne, VIC, Australia; ^4^ Department of Paediatrics, University of Melbourne, Parkville, VIC, Australia; ^5^ Melbourne Law School, University of Melbourne, Parkville, VIC, Australia; ^6^ Trans Health Research Group, Department of Medicine (Austin Health), University of Melbourne, Heidelberg, VIC, Australia

**Keywords:** transgender, gender non-binary, gender dysphoria, hormone replacement therapy, selective estrogen receptor modulators

## Abstract

Gender dysphoria describes the distress associated with having a gender identity that differs from one’s birth-assigned sex. To relieve this distress, transgender, and gender diverse (henceforth, trans) individuals commonly undergo medical transition involving hormonal treatments. Current hormonal treatment guidelines cater almost exclusively for those who wish to transition from male to female or vice versa. In contrast, there is a dearth of hormonal options for those trans individuals who identify as non-binary and seek an androgynous appearance that is neither overtly male nor female. Though prolonged puberty suppression with gonadotrophin releasing hormone agonists (GnRHa) could in theory be gender-affirming by preventing the development of unwanted secondary sex characteristics, this treatment option would be limited to pre- or peri-pubertal adolescents and likely have harmful effects. Here, we discuss the theoretical use of Selective Estrogen Receptor Modulators (SERMs) for non-binary people assigned male at birth (AMAB) who are seeking an androgynous appearance through partial feminization without breast growth. Given their unique range of pharmacodynamic effects, SERMs may represent a potential gender-affirming treatment for this population, but there is a lack of knowledge regarding their use and potentially adverse effects in this context.

## Introduction

Trans individuals have a gender identity that is different from the sex they were assigned at birth, as opposed to cisgender people, whose gender identity corresponds with their assigned sex (1). Although some trans individuals identify as either male or female, others have a non-binary gender identity that does not accord with either of these traditional options. Whilst non-binary is a gender identity in itself, it is also an umbrella term that encompasses any gender identity that is not exclusively male or female and may include people who identify as genderfluid, gender non-conforming, transmasculine, transfeminine or agender amongst others. Non-binary individuals in recent studies constitute more than 25% of the trans population (1–3).

Like trans people with binary identities, those with a non-binary gender identity frequently face social difficulties, including social exclusion at school/work and feelings of isolation. As a result, these individuals often experience poor mental health that is worse than those with binary identities (1, 4). In studies using self-report surveys, non-binary people were less likely to have family support, while significantly more likely to feel isolated, report poorer mental health, and consider suicide than trans people with binary identities (1, 2, 4, 5). Moreover, non-binary individuals frequently encounter a lack of understanding within society regarding their gender identity, including from health professionals (6).

A common reason for poor mental health in trans (including non-binary) individuals is gender dysphoria (GD), which is the distress associated with having a gender identity that differs from one’s sex assigned at birth (7). Non-binary people seeking assistance to reduce their GD now constitute a significant proportion (10-30%) of referrals to paediatric and adult gender clinics (1, 8). Given the growing population of non-binary individuals seeking gender-affirming clinical care and the highly vulnerable nature of this population (9), it is important to have appropriate medical options to help them achieve a body they regard as consistent with their identity and to reduce their GD.

Medical treatment with gender-affirming hormones, such as testosterone and estradiol may be appropriate for some non-binary individuals who wish to masculinize or feminize respectively (10, 11). However, other non-binary individuals may seek an androgynous body that is neither overtly male nor female, and there is a lack of medical treatment options specifically tested or approved for use in this group to prevent the development of secondary sex characteristics on an ongoing basis (12). In this article, we therefore consider Selective Estrogen Receptor Modulators (SERMs) as a potential treatment option for non-binary people assigned male at birth (AMAB) who wish to partially feminize without undergoing breast development.

## Current Treatment Options for Non-Binary Trans People

Currently, medical transition options for trans people vary depending on an individual’s pubertal stage. For trans adults, treatment options to induce secondary sex characteristics consistent with a person’s gender identity include gender-affirming hormone therapy and surgical interventions (10, 11). Gender-affirming hormone therapy may involve testosterone for trans people assigned female at birth (AFAB) and estradiol and anti-androgens for trans people AMAB (10, 11). For trans adolescents in early/mid puberty, gonadotropin-releasing hormone agonists (GnRHa), commonly known as “puberty blockers”, may be given to suppress puberty before or in conjunction with the use of gender-affirming hormone treatment (10, 11, 13, 14). The effects of GnRHa are considered reversible insofar as stopping treatment allows puberty to resume (10, 11, 13, 14). While many effects of testosterone and estradiol are reversible, some including voice deepening and breast development are not (10, 13). The availability of irreversible surgical interventions for adolescents depends on the age and needs of the individual. Individuals in some jurisdictions are able to access chest reconstructive surgery but rarely genital surgery before the age of 18 years (13).

Under current treatment schema, non-binary people who wish for an androgynous body have limited treatment options. For those AFAB, low-dose testosterone is being used empirically with the aim of reducing feminine features by helping to re-distribute adipose tissue and deepen the voice (12). Such effects may be difficult to selectively titrate while avoiding other potentially unwanted masculinizing changes such as facial hair growth. In addition, chest dysphoria is regularly experienced by trans individuals AFAB, and chest reconstructive surgery can enable removal of unwanted breast tissue for those who have progressed through puberty (13). Those AMAB may choose to use low-dose estradiol to partially feminize, though the widespread effects of estradiol could potentially cause unwanted feminising changes, such as breast growth (12). For non-binary adolescents who present in early puberty, long-term GnRHa use may provide a theoretical solution to maintain an androgynous appearance without any secondary sex characteristics (15). However, such a strategy carries substantial risks as described below, which limits its use for this purpose.

## Maintaining Androgyny *via* Long-Term Puberty Suppression

Pubertal suppression with GnRHa blocks the release of luteinizing hormone (LH) and follicle-stimulating hormone (FSH) from the pituitary gland, preventing production of testosterone and estradiol from the testes and ovaries respectively (16). Without testosterone or estradiol, sex-specific secondary sex characteristics, such as breast development in those AFAB or voice deepening in those AMAB, do not progress (16). In this way, for many trans adolescents, puberty blockers can help prevent an exacerbation of GD and the attendant anxiety that often accompanies typical progression through puberty (17).

However, since the pubertal increase in either testosterone or estradiol levels is important for the development and function of other body systems, use of GnRHa in trans adolescents can also cause unwanted outcomes. For example, pubertal sex hormone is essential for healthy bone development, with testosterone and estradiol acting as key promoters of new bone deposition and increased bone mass (18). Consistent with this, use of GnRHa in trans adolescents is associated with reduced bone mineral density (BMD) (19, 20), and the potential functional impact of this on bone quality and fracture risk in adulthood remains unknown (21). Long-term GnRHa therapy from early puberty would also be expected to inhibit typical pubertal development of reproductive potential (15, 22); this effect is considered likely reversible but has not been directly studied (22). Finally, significant brain and cognitive development occurs during adolescence. Although it is unclear to what extent pubertal hormones positively influence this development, it stands to reason that puberty suppression might impact this process (16, 17, 21).

For trans adolescents who receive puberty blockers and wish to subsequently feminize or masculinize their bodies, use of gender-affirming hormones is likely to ameliorate some of these concerns associated with GnRHa use, though further study is required (14, 23). However, for non-binary adolescents who wish to undertake *long-term* puberty suppression to maintain an androgynous body, these risks will presumably be exacerbated. Although such an approach might be ethically justified in some circumstances (15, 24), most physicians would be unlikely to prescribe puberty blockers as independent, long-term treatment (25). In addition, this treatment would not be effective for non-binary individuals who have completed puberty. Having alternative therapeutic options in such cases would therefore be valuable.

## SERMs: A Possible Solution?

### Overview

SERMs are chemically diverse compounds that bind to alpha or beta subunits of the estrogen receptor and produce both agonistic (i.e., estrogenic) and antagonistic (i.e., anti-estrogenic) effects within different cell types and tissues (**Table 1**) (23, 43). With the advent of newer drug designs in recent decades, SERMs are categorized into three generations. In general, each generation has estrogenic and anti-estrogenic effects on different parts of the body. In reality, many effects are drug-specific, still debated, or unknown (23). Across the first, second and third generations of SERMs, the most popular approved treatments are tamoxifen, raloxifene (RLX), and lasofoxifene respectively (29). Tamoxifen is most commonly used to treat pre-menopausal breast cancer (23). RLX is widely approved for post-menopausal osteoporosis (35). Lasofoxifene has been approved in the EU for both osteoporosis and vaginal atrophy (45) and is currently being reviewed by the FDA as a treatment for breast cancer (48).

**Table 1:** Differential effects of GnRHa, SERMs, and Estrogen.

Treatment Type	Breast Tissue	Vertebral Bones	Non-Vertebral Bones	Body Composition/Gynoid Fat Distribution	Skin	DVT Risk
**GnRHa**	– (13, 21)	– (19–21)	– (19–21)	MR (21, 26)	UN	+ (27)
**Estradiol**	+++ (28)	+++ (28, 29)	+++ (28, 29)	+ (30)	++ (31–33)	+ (28, 34)
**Tamoxifen**	– (29, 35)	+ (29)	+ (35, 36)	UN	– (33)	++ (29, 35, 37)
**(SERM Gen 1)**						
**Raloxifene**	– (29, 35)	++ (29, 35, 38)	0 (29, 35, 38, 39)	+ (40, 41)	+ (31, 32)	++ (35)
**(SERM Gen 2)**						
**Lasofoxifene**	– (35, 42)	+++ (43, 44)	++ (35, 42, 45)	UN	UN	++^*^ (35, 42, 46)
**(SERM Gen 3)**						

–, antagonistic effect; 0, neutral effect; +, mild agonistic effect; ++, moderate agonistic effect; +++, major agonistic effects; UN, unclear/unknown; MR, mixed results.

^*^Literature indicates increased DVT risk but lowered risk of heart disease and stroke associated with lasofoxifene (26, 47).

### Theoretical Use of SERMs as a Treatment for Non-Binary Individuals AMAB

Given their tissue-specific antagonistic and agonistic effects, SERMs could potentially be suitable either as an independent or combined treatment modality for the gender-affirming care of non-binary individuals AMAB who desire partial feminization without breast growth. Specific SERMs (**Table 1**) have been shown to inhibit breast and endometrial tissue growth, while exhibiting beneficial estrogenic effects on bones in pre- and post-menopausal cisgender women (35, 43). These effects are not well-studied in people AMAB. Theoretically, a SERM with anti-estrogenic effects on breast but estrogenic effects on bone, fat composition, and skin might offer appeal as a hormone treatment for a non-binary person AMAB who desires an androgynous body.

While SERMs could be used independently for non-binary adults AMAB to induce partial feminization, SERMs alone would not be expected to block unwanted pubertal masculinization in non-binary adolescents AMAB. Androgen blockade would still be required in this younger population. In the following sections, we explore the theoretical use of SERMs in this context, extrapolating from what is known about their use in other situations.

### SERMs May Promote Feminine Body Shape and Changes in Skin

Similar to estradiol hormone treatment, SERMs have been found to promote changes in body composition and skin that may be desired by non-binary people AMAB. In post-menopausal cisgender women, studies have found RLX to be an estrogen receptor agonist in adipose tissue, promoting shifts from android to gynoid fat patterns (40) and increasing fat-free mass and total body water (41). In this way, RLX may contribute to a more feminine body shape. In other studies of post-menopausal cisgender women, RLX has been shown to increase skin elasticity (31) and stimulate collagen biosynthesis (32). Thus, RLX is likely to promote softer, more stereotypically feminine skin. Though these changes may assist in the partial feminization of a non-binary individual AMAB, the effects of SERMs on skin and body composition are not widely studied outside of the population of post-menopausal cisgender women and further research is needed.

### SERMs Can Inhibit Breast Tissue Growth

Most SERMs inhibit the effects of estradiol on breast tissue growth and consequently are used to treat estradiol-responsive breast cancers (23, 49). Historically, tamoxifen has been the most commonly utilized SERM for treatment of metastatic, estrogen receptor-positive (ER+) cancers, where it is effective in lowering recurrence and mortality rates (23). Though not as commonly used for breast cancer treatment, newer SERMs such as RLX are also anti-estrogenic in breast tissue and deter breast cancer growth (35).

Consistent with their anti-estrogenic effect on breast tissue, SERMs such as tamoxifen have been reported as an effective alternative to surgery to treat persistent gynaecomastia in cisgender adolescent and adult males (49, 50). They have also been shown to improve symptoms associated with juvenile breast hypertrophy in adolescent cisgender females (51, 52). Although these studies involved small numbers of patients, they suggest that SERMs could help avoid breast growth in non-binary people AMAB who desire some feminization without breast development.

### SERMs and Bone Health

While promoting partial feminization without breast growth, SERMs have pro-estrogenic effects on bones and could also theoretically mitigate negative bone effects associated with GnRHa and/or anti-androgen use in non-binary adolescents and adults AMAB. A variety of SERMs have now been approved for treating osteoporosis in adults (23, 43) based upon their ability to improve BMD and/or fracture risk by up to 55% (39) in post-menopausal cisgender women. However, whether this occurs in people AMAB who may also be androgen deficient is unknown.

Though tamoxifen, RLX and lasofoxifene have all been associated with increases in BMD in post-menopausal cisgender women, there may be important differences between different generation SERMs in terms of bone health. For instance, only RLX and lasofoxifene have shown fracture benefit. Moreover, while lasofoxifene reduces the incidence of both vertebral and non-vertebral fractures (45), RLX was found to reduce the incidence of vertebral but not non-vertebral fractures (38, 39). It should also be noted that the reduction of fracture risk (particularly at the hip) for SERMs is less than that of bisphosphonates. As such, SERMs are frequently reserved for use in osteoporosis under specific scenarios (i.e. for younger post-menopausal cisgender women or those with a family history of invasive breast cancer) (43).

Importantly, the positive bone health effects of SERMs in post-menopausal cisgender women cannot be directly extrapolated to gender non-binary young people AMAB. Post-menopausal cisgender women have already attained peak bone mass (PBM) in early adulthood (53), and it is the physiological estradiol deficiency that causes post-menopausal declines in BMD (54). In contrast, adolescence is associated with a significant increase in BMD directly resulting from effects of pubertal hormones. Adolescents exhibit an average annual accrual of 6-10% and an overall doubling from childhood through the mid 20s (55). Hence, use of SERMs to potentially promote bone health in trans adolescents AMAB would be physiologically distinct from their use in post-menopausal cisgender women. Specifically, while the latter seeks to maintain BMD that has already peaked and has strong supporting evidence, the former aims to promote the attainment of PBM which is a context in which SERMs are untested. Thus, it is unclear to what extent – if at all – SERMs increase BMD during adolescence and how PBM would be affected.

Indeed, there is evidence that questions the extent of SERMs’ pro-estrogenic effects on bones. For example, in a three-year prospective study of premenopausal cisgender women treated with tamoxifen for breast cancer, the average annual lumbar spine BMD fell 1.4%, compared to a 0.2% increase in those who received a placebo. These results prompted the authors to conclude that tamoxifen can act as a partial competitive antagonist of endogenous estradiol to cause increased bone remodelling (56). Similarly, another study on post-chemotherapy tamoxifen usage in cisgender women with breast cancer found that premenopausal patients experienced a 4.6% decrease in their baseline BMD compared to a 0.6% gain in BMD in those not on tamoxifen. However, it should be noted that this result may have been confounded by the fact that those who received tamoxifen were from the outset inherently different to those who did not (based on hormone receptor status of their tumors) (57). While both of these studies focused on tamoxifen use in adults with breast cancer, if similar findings held true in healthy adolescents with SERMs, the use of SERMs might in fact be detrimental to the bone health of trans young people.

Hence, caution is required. With no studies having assessed bone outcomes in trans people using SERMs, further research is required, including in those AMAB. Thus far, pre-clinical data has shown that lasofoxifene prevented bone loss by inhibiting bone turnover related to aging and low serum testosterone levels in male rats (58). In addition, a study of cisgender men with prostate cancer found that RLX prevented harmful effects of GnRHa on bone, including bone loss (59). While the use of SERMs in this context might arguably be the most directly analogous to their potential use in trans people AMAB, clearly further assessment of the skeletal effects of SERMs in adolescents and adults AMAB are required.

### Effects of SERMs on Other Body Systems

Given that significant brain maturation occurs during adolescence, testosterone and estradiol may potentially facilitate adolescent brain development and functioning. It is unknown whether the absence of sex steroids secondary to GnRHa use in trans adolescents has any cognitive effects (17). Similarly, the effects of SERMs on brain development and function are uncertain. *In vitro* studies have suggested that SERMs promote synaptic transmission, increase axonal growth, and facilitate the regeneration of damaged synapses (60, 61). *In vivo* use of tamoxifen in animal models and cisgender women with breast cancer is associated with improved cognitive function (62, 63). The relevance of such findings for the use of SERMs in non-binary individuals AMAB is unclear.

Among cisgender males, lower sex hormone concentrations in adulthood have also been associated with an increased risk of cardiovascular disease (64, 65). Interestingly, a recent study of >8000 60-80 year-old post-menopausal cisgender women receiving daily lasofoxifene showed a 32% reduction in major coronary events over five years compared to hypogonadal post-menopausal cisgender women (45). While these findings suggest that SERMs might promote cardiovascular health, such effects again cannot be directly extrapolated to non-binary people AMAB, given likely different mechanisms underlying their cardiovascular risk profile.

### Health Risks of SERMs

Common side effects of SERMs include hair loss, nausea, mood disturbance, and hot flashes (23, 43). In addition, long-term use of SERMs have been associated with several other risks, all of which are important to consider when balancing their potential use as a gender affirming treatment.

First, as previously observed with estrogen replacement therapy for postmenopausal cisgender women (34), an increased risk of thromboembolic events has been associated with SERM use. RLX has been shown to increase the risk of deep vein thrombosis by up to 54% and the risk of pulmonary embolism by 91% (66). Similar data is available for tamoxifen (37). Whether the rates of stroke and myocardial infarctions are also increased remains unclear, with previous studies showing inconsistent results (29, 67, 68).

Secondly, long-term SERM use has been associated with hypertriglyceridemia and hepatic steatosis (69), as well as altered glucose metabolism and increased risk of diabetes (70). In a 12-year longitudinal study involving over 22,000 cisgender women with breast cancer, a 31% increased diabetes risk was observed in tamoxifen users versus non-users (71). Notably, the majority of SERM-based metabolic studies have been conducted on cisgender women with and without breast cancer (69). Whether similar metabolic effects would occur in those AMAB is unclear.

Finally, SERM use can increase testosterone production in those AMAB, owing to their antagonistic actions at the estrogen receptor within the hypothalamus and pituitary. Specifically, tamoxifen was found to increase serum FSH, LH and testosterone levels in cisgender men by less than 2-fold, while raloxifene showed only minimal increase (47). The clinical relevance of such changes is unclear but, may cause or further exacerbate unwanted masculinization.

## Clinical Implications

Whilst SERMs should not be routinely recommended as treatment options, clinicians may encounter scenarios where SERMs may be requested by patients AMAB who desire feminization of skin or fat distribution without breast growth. Indeed, we are aware of SERMs already being used anecdotally by adults in this context. For patients wanting to use SERMs to affirm their gender, the lack of evidence for efficacy or safety must be highlighted, and there should be a clear explanation that treatment is experimental. To provide informed consent, individuals need to understand the selective differential effects of SERMs, their potential lack of benefit, and the risk of adverse effects. If a shared decision between patient and doctor is made to trial the use of a SERM, then clear goals of treatment (e.g., fat redistribution) over a pre-specified timeline (e.g., 3-6 months) should be determined. If treatment goals are not achieved, treatment should be ceased. Given Raloxifene is the only SERM with evidence for agonistic effects on fat and body composition (40, 41), it would appear to be the most suitable choice at present and would ideally be used in the context of a prospective research study. Consistent with this, clinical trials – ideally across multiple centers to aid in patient recruitment – are required to provide greater evidence regarding the potential benefits and harms of using SERMs in non-binary individuals.

## Conclusion

In the clinical setting, increasing numbers of non-binary individuals are seeking gender-affirming hormone therapy, and some desire androgynous physical characteristics to align with their gender identity. This can pose challenges in tailoring treatment options, and a lack of well-defined therapeutic pathways leaves non-binary people at risk of persistent GD and ongoing vulnerability to adverse mental health outcomes. SERMs may be a theoretically attractive option for non-binary people AMAB who desire partial feminization without breast growth. However, there is a dearth of evidence regarding the safety and efficacy of SERMs in this context. An individualised, shared decision-making approach to care is therefore needed when considering the use of SERMs in non-binary people, with acknowledgement of their experimental nature, significant potential risk and need for further research.

## Data Availability Statement

The original contributions presented in the study are included in the article/supplementary material. Further inquiries can be directed to the corresponding author.

## Author Contributions

JX performed the relevant literature review, screened studies for inclusion, extracted relevant information from the included articles, drafted the initial manuscript, and revised the manuscript. KP conceptualised the manuscript and, together with LN, AC, MO’C, and SZ reviewed and revised the manuscript. All authors contributed to the article and approved the submitted version.

## Funding

KP is supported by fellowships from the Royal Children’s Hospital Foundation and Hugh Williamson Trust Foundation. AC is supported by an Australian Government National Health and Medical Research Council Early Career Fellowship (#1143333) and receives support from the Viertel Charitable Foundation Clinical Investigator Award, Endocrine Society of Australia Ken-Wynne Award and Royal Australasian College of Physicians Foundation. MO’C, LN, and KP acknowledge the infrastructure funding received from the Victorian State Government through the Operational Infrastructure Support (OIS) Program.

## Conflict of Interest

The authors declare that the research was conducted in the absence of any commercial or financial relationships that could be construed as a potential conflict of interest.

## Abbreviations

AFAB, assigned female at birth; AMAB, assigned male at birth; GnRHa, gonadotrophin releasing hormone agonists; SERM, selective estrogen receptor modulator.
